# Presence of Viable Bacilli in Multibacillary Leprosy After 12 Doses of Multidrug Therapy

**DOI:** 10.1590/0037-8682-0112-2025

**Published:** 2025-12-15

**Authors:** Danyenne Rejane de Assis, Josafá Gonçalves Barreto, Marcio Cesar Reino Gaggini, Amílcar Sabino Damazo, Cor Jesus Fernandes Fontes, Marcos César Florian

**Affiliations:** 1 Universidade Federal Mato Grosso, Hospital Universitário Júlio Müller, Empresa Brasileira de Serviços Hospitalares, Cuiabá, MT, Brasil.; 2 Universidade Federal do Pará, Laboratório de Epidemiologia Espacial, Castanhal, PA, Brasil.; 3 Universidade Brasil, Fernandópolis, SP, Brasil.; 4 Universidade Federal de Mato Grosso, Cuiabá, MT, Brasil.; 5 Universidade Federal de São Paulo, Escola Paulista de Medicina (EPM), São Paulo, SP, Brasil.

**Keywords:** Multibacillary leprosy, Mycobacterium leprae, Multidrug therapy

## Abstract

**Background:**

We investigated the clinical profiles of patients with multibacillary leprosy treated with at least 12 doses of multidrug therapy (MDT), in whom intact *Mycobacterium leprae* bacilli were identified in skin biopsies.

**Methods:**

Clinical and therapeutic characteristics of 30 patients were analyzed, and their association with bacillary integrity was assessed.

**Results:**

Leprosy reactions were commonly observed. Two-thirds of patients completed treatment within 5 years. A strong association was observed between the 12-dose regimen and the presence of intact bacilli.

**Conclusions:**

Clinical and laboratory aspects should be considered when deciding to discontinue MDT, particularly if leprosy reactions occur.

Leprosy, a chronic and neglected infectious disease caused by *Mycobacterium leprae* or *Mycobacterium lepromatosis*, has existed for thousands of years; however, many aspects of its pathogenesis and clinical management remain incompletely understood. The host immune response largely determines clinical and histopathological manifestations^1^. Leprosy reactions, which may occur before, during, or after treatment, are classified into two main types: type 1 reaction (T1R)/reversal reaction and type 2 reaction (T2R)/erythema nodosum leprosum (ENL)^1^ ^,^ ^2^.

Since the 1980s, the World Health Organization has recommended multidrug therapy (MDT) as the standard treatment for leprosy. This therapy combines rifampicin, dapsone, and clofazimine, administered over 12 months for multibacillary cases, as recommended by the Brazilian guidelines^3^ ^,^ ^4^. Despite the widespread introduction of MDT, considerable challenges in leprosy treatment remain: standardized parameters to confirm disease resolution are lacking, and patient discharge is primarily based on the completion of the MDT regimen^3^ ^-^ ^5^.

In addition to the bacillary index (BI), the morphological index (MI)-obtained through bacilloscopy of slit-skin smears or skin biopsies-can serve as a valuable tool for assessing disease resolution by classifying *M. leprae* morphology as intact, fragmented, or granular. Intact bacilli indicate viable forms. Histological and bacilloscopic examination of skin biopsies provides a more reliable indicator of bacillary viability than slit-skin smears^6^ ^,^ ^7^.

Given the potential for disease progression to disabilities and the risk of persistent transmission^7^, identifying the clinical and therapeutic factors associated with persistent disease activity following standard MDT is crucial. In this study, we evaluated the clinical profiles of patients treated for multibacillary leprosy who had completed at least 12 doses of MDT and exhibited intact bacilli in skin biopsy, providing insights into the management of challenging cases after MDT completion.

This cross-sectional, retrospective study was conducted by analyzing medical records from the Leprosy Outpatient Clinic of the Júlio Müller University Hospital (HUJM), which is part of the Brazilian Hospital Services Company (EBSERH) network and affiliated with the Federal University of Mato Grosso (UFMT). Cases were clinically classified based on the Madrid Classification (1953) during the initial routine evaluation. The study was approved by the Research Ethics Committee under certificate number 67294623.6.0000.5541 and authorization number 5933798. All participants provided written informed consent.

We included 30 patients with intact bacilli in skin biopsies who had previously received at least 12 doses of MDT, including some patients who were still undergoing treatment. Skin biopsies were performed at the discretion of the physician when disease activity was suspected. Bacilloscopic analysis of skin biopsies, including MI, was performed between November 2020 and December 2022 at the UFMT Histology Laboratory using the Fite-Faraco staining method. As MI analysis requires a high level of expertise and is inherently subjective^8^, only patients with ≥ 50% intact bacilli were considered.

Bacilloscopy of slit-skin smears was performed either at the hospital laboratory or via the accredited network of the Central Public Health Laboratories (LACEN) using the standard Ziehl-Neelsen staining technique; BI was scored based on the Ridley classification^4^. Slit-skin smears were not available for all patients, especially those originally treated at other centers.

To investigate antimicrobial resistance, skin biopsy samples were sent to the Lauro de Souza Lima Institute in Bauru, São Paulo, Brazil, when logistically feasible. Resistance testing was performed using polymerase chain reaction (PCR) to detect mutations in target genes associated with resistance to rifampicin (*rpoB*), ofloxacin (*gyrA*), and dapsone (*folP1*)^4^.

Data were entered into an Excel spreadsheet and analyzed using the "Social Science Statistics" (https://www.socscistatistics.com/, accessed August 2023) and "OpenEPI" digital (version 3.01, updated April 6, 2013; https://www.openepi.com/Menu/OE_Menu.htm, accessed August 2023) platforms. Continuous variables were expressed as means with standard deviations (SDs) or medians, whereas categorical variables were presented as absolute and relative frequencies (percentages). Variables were analyzed and compared based on the degree of bacillary integrity (≥ 75% and < 75%). Statistical analyses included the chi-square test, Fisher’s exact test, Mann-Whitney test, and Pearson correlation. A significance level (alpha) of 0.05 was assumed for all comparative analyses.

Thirty patients were included, all classified as having dimorphic leprosy, with all reporting adherence to treatment; 19 (63.3%) were male. The mean age was 53 years (median, 50 years; range, 26-84). Most patients (21; 70%) had comorbidities (Table 1), with systemic arterial hypertension being the most common (33.3%), alone or in combination with other conditions. Other comorbidities included type 2 diabetes mellitus, hypothyroidism, cardiomyopathy, chronic kidney disease, HIV/AIDS infection with viral suppression, alcohol consumption, smoking, depression, osteoporosis, fibromyalgia, vasculitis, Sjögren’s syndrome, latent syphilis, and asthma.

**TABLE 1:** Characteristics of 30 patients with intact bacilli on skin biopsies after receiving at least 12 doses of MDT, with biopsies performed between November 2020 and December 2022 at HUJM-UFMT/EBSERH**.**

	N (30)	%
Age (median, min., and max.)	50 (26; 84)	-
Male	19	63.0
Comorbidities	21	70.0
Borderline form	30	100
T2R (ENL) episodes	8	26.7
T1R episodes	10	33.3
Simultaneous T1R and T2R episodes	3	10
Ongoing treatment	3	10
One previous treatment	24	80.0
two or more previous treatments	3	10.0
Initial standard MDT	30	100
Dapsone replaced due to AE	3	10.0
12-dose regimen*	17	56.7
24-dose regimen	13	43.3
Use of corticosteroids	20	66,7
MDT completed < 1 year before biopsy	7	23,3
MDT completed 1‒5 years before biopsy	12	40.0
MDT completed > 5 years before biopsy	8	26.7
Average BI in slit-skin smear	2.3	-
Average BI in skin biopsy	3.1	-

*Includes the two patients who received 13 and 14 doses. MDT: multidrug therapy; **AE:** adverse event; **BI:** bacillary index.

Thirteen (43.3%) patients received 24-dose MDT because they showed signs of therapeutic insufficiency after the initial 12-dose MDT^2^. Fifteen (50.0%) patients received 12-dose MDT, while two patients were still undergoing treatment (one on the 13th dose and one on the 14th dose). Indications for skin biopsy included ENL in 11 patients (36.7%), neural symptoms in five (16.6 %), T1R in one (3%), new or worsening skin lesions in four (13.8%), cutaneous neural symptoms in eight (26.6%), and isolated neural symptoms in six (20%). Biopsy sites comprised erythema nodosum nodules in eight patients (27.6%), macular lesions in 16 (53.3%), neural pathways in two (6.7%), unspecified sites in three (10%), and plaque lesions in one (3.4%). 

The median time between MDT completion and skin biopsy collection was 21 months (range: 0‒156 months). Most patients (12; 40%) had completed treatment between one and five years before inclusion, ten patients (33.3%) had completed treatment within the past year or were still undergoing treatment (three patients; 10%), and eight (26.7%) had completed > five years ago. Four patients (13.3%) had previously undergone multiple full treatment courses, one of whom was still receiving MDT at the time of the study (Table 1).

A baseline BI from slit-skin smears was available for 16 patients (53.3%), with a mean of 2.3; seven patients (43.7%) had a BI < 2, and nine (56.3%) had a BI ≥ 2. All patients were initially treated with uniform MDT. In three cases (10%), dapsone was substituted with ofloxacin (Table 1). The frequency of adverse events observed was comparable to that in an Indian study conducted at a tertiary care hospital, in which 12.3% of 171 MDT recipients experienced side effects^9^. In our study, MDT-related adverse events included anemia in three cases (11.5%), associated methemoglobinemia in one (3.8%), and gastritis in one (3.8%). 

Leprosy reactions were documented in 21 (70 %) patients. Of these, eight (26.7%) developed T2R (one with reactive hands and feet and another with ulcerated erythema nodosum), three (10%) experienced mixed reactions (one with polymorphic erythema and reactive hands and feet), and ten (33.3%) had T1R, one of whom presented with neuritis only. Symptoms of neuritis were observed in 17 patients (56.7 %). Systemic corticosteroids were administered to 20 patients (66.7%) (Table 1), with seven (23.3%) receiving continuous therapy, 12 (40%) completing at least two cycles of treatment, and one (3.3%) completing a single cycle before intermittently self-administering low doses. The use of corticosteroids to manage leprosy reactions is well documented but carries the risk of significant side effects, as observed in this study^10^. Corticosteroid-induced diabetes occurred in one patient (5%), vertebral fracture due to osteoporosis in one patient (5%), and cataracts in one patient (5%).

The BI in skin fragments ranged from 1 to 6 (mean = 3.1), with values above 2 observed in 18 patients (60.0%) (Table 1). Regarding the MI, 16 patients (53.3%) had 100% intact bacilli in their biopsies, one (3.3%) had 75%, and 13 (43.3%) had between 50% and 70%. No patients had granular bacilli. At the time of skin biopsy, 20 patients had BI data from slit-skin smears (mean = 1.2), with only six patients (30%) having a BI > 2. A moderate positive correlation was observed between the two indices (r=0.51; p=0.022) (Figure 1). Bacilli were present in the biopsies of 10 patients (50%) who had negative slit-skin smears. Among patients with available slit-skin smear BI data both before and after treatment, the mean value decreased from 2.3 to 1.5.

**FIGURE 1:**
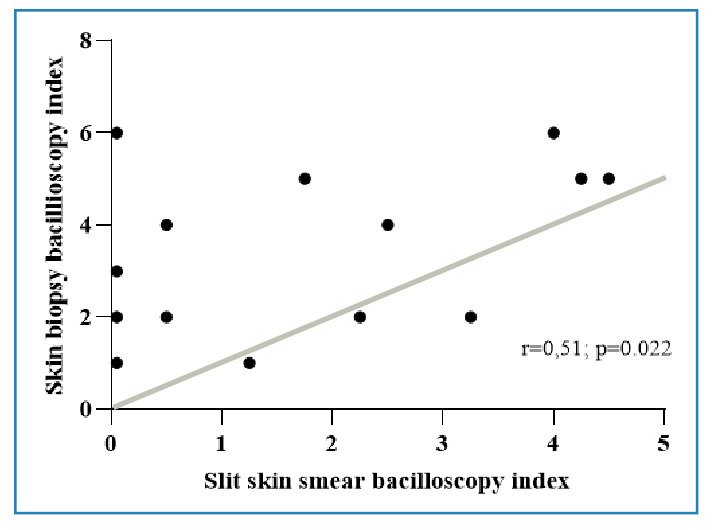
Correlation between slit-skin smear and skin biopsy bacillary index (November 2020‒December 2022).

Resistance testing was performed in 12 patients (40%). In one patient (8.3%), the result was inconclusive, and in another (8.3%), DNA was not detectable. Eight (66.7%) patients showed no resistance, whereas two (16,7%) exhibited resistance-one to dapsone and the other to ofloxacin; both were women with T1R and lesion enlargement. The rate of resistance observed in this study was markedly higher than the 1.2% reported by the National Resistance Surveillance system^11^. This elevated rate can be attributed to the fact that the sample consisted of complex cases referred to a tertiary medical center. However, drug resistance alone does not fully explain poor disease control in this group. 

The frequency of leprosy reactions in this study was higher than that reported in the literature, occurring in over two-thirds of patients. T1R occurs in approximately one-third of borderline leprosy cases, whereas ENL occurs in 10% of cases, possibly reaching up to 50% in lepromatous forms^1^. We hypothesized that the immunosuppressive effects of corticosteroids used to treat the reactions may have contributed to the persistence of bacilli and hindered disease healing in this study. However, no statistically significant difference in MI was observed between patients who received corticosteroids (65% having ≥ 75% intact bacilli) and those who did not (40%) (p =0.362), possibly due to the small sample size (Table 2). Similarly, a larger study conducted in India found no significant differences in outcomes between patients receiving corticosteroids and controls^12^.

**TABLE 2:** Distribution of patients based on the morphological index of skin biopsies obtained between November 2020 and December 2022 at HUJM-UFMT/EBSERH.

Characteristics	Intact Bacilli ≥ 75%	Intact Bacilli < 75%	p-value
12-dose MDT	12 (70.6%)	5 (29.4%)	0.039
24-dose MDT	5 (38.5%)	8 (61.5%)	
Months between MDT completion and biopsy (SD)	23.5 (28.4)	58.8 (50.8)	0.051
Leprosy reactions	12 (57.1%)	9 (55.6%)	0.653
Use of corticosteroids	13 (65%)	7 (35%)	0.362
No corticosteroids	4 (40%)	6 (60%)	

**MDT:** multidrug therapy.

The time from treatment completion to skin biopsy was not significantly associated with differences in MI. This interval was 23.5 months (SD =28.4) for patients with ≥ 75% intact bacilli and 58.8 months (SD =50.8) for those with < 75% (p =0.051) (Table 2). Bacillary clearance is a dynamic process that continues even after treatment; therefore, this parameter cannot be reliably used to assess ongoing disease activity^4^, which may explain the lack of a significant association in this analysis. The occurrence of reactions was not significantly higher in patients with higher bacillary integrity (57.1% vs. 55.6%, p = 0.623). The 12-month MDT regimen was the only factor associated with a higher percentage of intact bacilli (70.6% vs. 38.5% in patients who received the 24-month MDT; p = 0.039) (Table 2).

In this study, the patient profile was characterized by dimorphic forms in all cases, good self-reported treatment adherence, a relatively high median age, and a diverse spectrum of symptoms and comorbidities. There was a high prevalence of leprosy reactions and extensive use of corticosteroids. MDT was generally completed within 5 years before skin biopsy collection, and drug resistance was observed in 2 of 12 cases analyzed. The presence of a BI > 2 in the initial slit-skin smear in many patients indicated more advanced disease and/or impaired immune control. 

The high proportion of patients with intact bacilli in skin biopsies after MDT, along with the association between the 12-month regimen and a higher percentage of intact forms, underscores the challenge of discharging patients with leprosy based solely on treatment duration. These findings reinforce the need for clinical trials to re-evaluate cure criteria following fixed-duration therapy, especially for patients with high BIs or those who develop reactions. Similar concerns were reported in an Indian study, which quantified mRNA targets (16SrRNA, esxA, and hsp18) using quantitative PCR in skin samples from 36 patients before and after 12 months of MDT; increased expression was observed in 66.6%, 61.1%, and 55.5% of patients, respectively, indicating viable bacilli at treatment completion^13^.

This study had some limitations, including its observational design, absence of a control group, small sample size, and lack of bacillary viability testing using techniques such as mRNA-based qPCR, mouse footpad inoculation, or ATP measurement^13^ ^,^ ^14^. Data on contact evaluations were also limited, and re-exposure to *M. leprae* may have contributed to persistence or recurrence^4^. Despite these constraints, the results highlight the complexity of post-treatment evaluation in leprosy, particularly for patients with high BIs and those who develop reactions^5^ ^,^ ^6^ ^,^ ^14^ ^,^ ^15^. Further studies, ideally through well-designed clinical trials, are warranted to better define strategies for confirming cure and guiding safe patient discharge.

## Data Availability

Research data is only available upon request.
